# Assessment of interactions between 205 breast cancer susceptibility loci and 13 established risk factors in relation to breast cancer risk, in the Breast Cancer Association Consortium

**DOI:** 10.1093/ije/dyz193

**Published:** 2019-10-12

**Authors:** Pooja Middha Kapoor, Sara Lindström, Sabine Behrens, Xiaoliang Wang, Kyriaki Michailidou, Manjeet K Bolla, Qin Wang, Joe Dennis, Alison M Dunning, Paul D P Pharoah, Marjanka K Schmidt, Peter Kraft, Montserrat García-Closas, Douglas F Easton, Roger L Milne, Jenny Chang-Claude, on behalf of Breast Cancer Association Consortium

**Affiliations:** 1 Division of Cancer Epidemiology, German Cancer Research Center (DKFZ), Heidelberg, Germany; 2 Faculty of Medicine, University of Heidelberg, Heidelberg, Germany; 3 Department of Epidemiology, University of Washington School of Public Health, Seattle, WA, USA; 4 Public Health Sciences Division, Fred Hutchinson Cancer Research Center, Seattle, WA, USA; 5 Centre for Cancer Genetic Epidemiology, Department of Public Health and Primary Care, University of Cambridge, Cambridge, UK; 6 Department of Electron Microscopy/Molecular Pathology and Cyprus School of Molecular Medicine, Cyprus Institute of Neurology & Genetics, Nicosia, Cyprus; 7 Centre for Cancer Genetic Epidemiology, Department of Oncology, University of Cambridge, Cambridge, UK; 8 Division of Molecular Pathology, The Netherlands Cancer Institute - Antoni van Leeuwenhoek Hospital, Amsterdam, The Netherlands; 9 Division of Psychosocial Research and Epidemiology, The Netherlands Cancer Institute - Antoni van Leeuwenhoek Hospital, Amsterdam, The Netherlands; 10 Program in Genetic Epidemiology and Statistical Genetics, Boston, MA, USA; 11 Department of Epidemiology, Harvard TH Chan School of Public Health, Boston, MA, USA; 12 Division of Cancer Epidemiology and Genetics, National Cancer Institute, Bethesda, MD, USA; 13 Division of Genetics and Epidemiology, Institute of Cancer Research, London, UK; 14 Cancer Epidemiology Division, Cancer Council Victoria, Melbourne, VIC, Australia; 15 Centre for Epidemiology and Biostatistics, Melbourne School of Population and Global Health, The University of Melbourne, Melbourne, VIC, Australia; 16 Precision Medicine, Monash University, Clayton, VIC, Australia; 17 Cancer Epidemiology Group, University Cancer Center Hamburg (UCCH), Hamburg, Germany

**Keywords:** Gene-environment interaction, breast cancer, single nucleotide polymorphism, epidemiology, risk factors, Europeans

## Abstract

**Background:**

Previous gene-environment interaction studies of breast cancer risk have provided sparse evidence of interactions. Using the largest available dataset to date, we performed a comprehensive assessment of potential effect modification of 205 common susceptibility variants by 13 established breast cancer risk factors, including replication of previously reported interactions.

**Methods:**

Analyses were performed using 28 176 cases and 32 209 controls genotyped with iCOGS array and 44 109 cases and 48 145 controls genotyped using OncoArray from the Breast Cancer Association Consortium (BCAC). Gene-environment interactions were assessed using unconditional logistic regression and likelihood ratio tests for breast cancer risk overall and by estrogen-receptor (ER) status. Bayesian false discovery probability was used to assess the noteworthiness of the meta-analysed array-specific interactions.

**Results:**

Noteworthy evidence of interaction at ≤1% prior probability was observed for three single nucleotide polymorphism (SNP)-risk factor pairs. SNP rs4442975 was associated with a greater reduction of risk of ER-positive breast cancer [odds ratio (OR)_int_ = 0.85 (0.78-0.93), *P*_int_ = 2.8 x 10^–4^] and overall breast cancer [OR_int_ = 0.85 (0.78-0.92), *P*_int_ = 7.4 x 10^–5^) in current users of estrogen-progesterone therapy compared with non-users. This finding was supported by replication using OncoArray data of the previously reported interaction between rs13387042 (r^2^ = 0.93 with rs4442975) and current estrogen-progesterone therapy for overall disease (*P*_int_ = 0.004). The two other interactions suggested stronger associations between SNP rs6596100 and ER-negative breast cancer with increasing parity and younger age at first birth.

**Conclusions:**

Overall, our study does not suggest strong effect modification of common breast cancer susceptibility variants by established risk factors.

**Key Messages**

- The association between common breast cancer susceptibility loci and breast cancer risk is not strongly modified by established breast cancer risk factors.
- The combined effect of susceptibility loci and established risk factors is thus well described by a multiplicative model.
- We found one noteworthy gene-environment (G x E) interaction with overall and with estrogen-receptor-positive breast cancer risk, which was replicated, and two novel noteworthy G x E interactions with ER-negative breast cancer risk.
- In an independent dataset, we replicated two previously reported G x E interactions.

## Introduction

Breast cancer is a complex disease with both environmental and genetic factors contributing to risk. Well-established modifiable and non-modifiable environmental factors include age at menarche, parity, age at first birth, breastfeeding, body mass index (BMI), use of menopausal hormonal therapy (MHT) and alcohol consumption.^1–6^ In addition, high/moderate-risk gene mutations such as *BRCA1*, *BRCA2*, *TP53*, *ATM* and *CHEK2* increase the risk of breast cancer,^7–14^ as well as multiple common, low-risk single nucleotide polymorphisms (SNPs) discovered through genome-wide association studies (GWAS). Approximately 170 genome-wide significant breast cancer susceptibility loci have been identified, including the recently published 65 novel loci associated with overall breast cancer and 10 loci with estrogen receptor (ER)-negative breast cancer risk, identified through the OncoArray project.^15^^,^^16^

Estimation of any combined effect of genetic and environmental factors, including gene-environment (G x E) interactions, is considered to possibly improve breast cancer risk prediction, and hence identification of women at high risk for targeted prevention. However, development of these risk models depends on knowledge of the joint effects of genetic and environmental risk factors, in particular departures from a multiplicative model (that is, G x E interaction on relative risk scale).^17^ More importantly, G x E studies of individual susceptibility loci may also provide insight on potential underlying biological mechanisms that could mediate causal effects of a factor on risk of breast cancer.

Previous G x E interaction studies of breast cancer have reported nearly 30 potential G x E interactions, with little evidence of departures from the multiplicative model.^18^^,^^19^ Most reported G x E interactions for breast cancer have not been replicated in independent datasets. Two G x E interactions were replicated using data from the Breast Cancer Association Consortium (BCAC),^20^ but were not replicated in a smaller study by the Breast and Prostate Cancer Cohort Consortium.^21^ In this study, we assess interactions between 205 known common breast cancer susceptibility loci and 13 established environmental risk factors in relation to risk of overall and of ER-specific breast cancer for women of European ancestry, using the largest available dataset to date from the Breast Cancer Association Consortium (BCAC). Additionally, we attempted to replicate previously reported potential G x E interactions.^18^

## Methods

### Study population

We analysed data from 46 studies (16 prospective cohorts, 14 population-based case-control studies and 16 non-population based studies) participating in BCAC (Supplementary Table 1, available as Supplementary data at *IJE* online). Participants were excluded if they were male, were of non-European descent, had breast tumours of unknown invasiveness, or had *in situ* disease or prevalent disease at the time of assessment. Women with unknown age at reference date (defined as date of diagnosis for cases and of interview for controls) were also excluded. For each risk factor, only studies with risk factor information for at least 150 cases and 150 controls were included. All participating studies were approved by the relevant ethics committees and informed consent was obtained from study participants.

### Data harmonization and variable definition

Data for risk factors from different studies were harmonized according to a common data dictionary and were centrally quality controlled. For both case-control and cohort studies, epidemiological risk factor data were derived with reference to reference date (described above). We used reference age as surrogate to categorize women as probably premenopausal (<54 years) or postmenopausal (≥54 years) status. The environmental variables available for analysis were: age at menarche (per 2 years); ever parous (yes or no); for parous women, number of full-term pregnancies (1, 2, 3 and ≥4), age at first full-term pregnancy (per 5 years), ever breastfed (yes or no), duration of breastfeeding (per 12 months); and for all women, ever use of oral contraceptives (yes or no), adult body mass index (BMI) separately for pre- and postmenopausal women (per 5 kg/m^2^), adult height (per 5 cm), lifetime alcohol consumption (per 10 g/day), current smoking (yes or no) and current use of combined estrogen-progesterone menopausal hormonal therapy (MHT) (yes or no) as well as current use of estrogen-only MHT (yes or no) for postmenopausal women.

### Genetic data

Samples were genotyped using one of the two SNP arrays: iCOGS^22^ or OncoArray.^15^ Included in the analyses were 28 176 cases and 32 209 controls of European ancestry genotyped by the custom iSelect genotyping array (iCOGS), comprising 211 155 SNPs,^22^ and 44 109 cases and 48 145 controls genotyped using the OncoArray 500 K, comprising 533 000 SNPs, nearly 260 000 of which were selected as a ‘GWAS backbone’ (Illumina HumanCore).^23^ These data were used to impute genotypes for ∼11.8 M SNPs using the 1000 Genomes Project (phase 3 version 5) reference panel.^15^^,^^16^ Details of genotyping and quality control procedures for the iCOGS and OncoArray projects are described in more detail elsewhere.^15^^,^^22^^,^^23^

A total of 205 common breast cancer susceptibility variants were selected for evaluation of G x E interactions (Supplementary Table 2, available as Supplementary data at *IJE* online). These variants have been associated with breast cancer risk either through GWAS^24–34^ or by fine mapping of associated regions.^35–52^ Of these, 72 were identified through the OncoArray project and had not been previously evaluated for G x E interactions.^15^^,^^16^

For replication of the previously reported interactions, we analysed a subset of 30 544 cases and 37 616 controls genotyped using the OncoArray array, which had not been included in previous G x E studies. We evaluated 33 potential G x E interactions that had been previously reported (Supplementary Table 3, available as Supplementary data at *IJE* online).^18^

### Statistical analysis

Unconditional logistic regression analysis was employed to assess associations of SNPs and risk factors with breast cancer risk. For SNPs, the estimated number of minor alleles based on imputation was included as a continuous variable. SNP-risk factor interactions were assessed using likelihood ratio tests, based on unconditional logistic regression models with and without an interaction term between the SNP and risk factor of interest. All analyses were adjusted for study, reference age and 10 ancestry-informative principal components. To account for differential main effects of risk factors by study design, we included an interaction term between the risk factor of interest and an indicator variable for study design (population-based and non-population-based), along with the main effect for study design.

Analyses were conducted separately for overall breast cancer risk and for ER subtype-specific breast cancer risk. The analyses were performed separately for women genotyped by iCOGS or OncoArray, and the results were meta-analysed using a fixed-effects inverse-variance weighted model. Between-study heterogeneity in the G x E interaction effect estimates was assessed by Cochrane’s Q test and I^2^ index.

MHT was classified into estrogen-progesterone therapy (EPT) and estrogen-only therapy (ET). Models assessing the association with current MHT use by type were adjusted for former use of MHT and use of any MHT preparation other than the one of interest. All analyses of MHT use were restricted to postmenopausal women. Models evaluating the association with current smoking were adjusted for former smoking.

To assess the noteworthiness of the observed G x E interactions, we calculated Bayesian false discovery probability (BFDP) at five different prior probabilities for a true association (20%, 10%, 1%, 0.1% and 0.01%). G x E interactions with BFDP <80% were considered as noteworthy. This was based on the assumption of a 4-fold cost of a false non-discovery compared with the cost of a false discovery and that the probability of observing a true interaction odds ratio (OR) inside the range of 0.66-1.50 was 95%, as proposed by Wakefield *et al*.^53^ We also computed a complementary measure to BFDP known as approximate Bayes factor (ABF). This approximates the ratio of the probability of the data given that the null hypothesis is true to the probability of the data when the alternative hypothesis is true, the null hypothesis being absence of any interaction. Therefore, a lower ABF favours the alternative hypothesis over the null hypothesis of absence of an interaction. For noteworthy G x E interactions, we performed stratified analyses by categories of the environmental risk factor using logistic regression. Analyses were carried out using SAS 9.4 or R version 3.4.2. Meta-analyses and tests of between-study heterogeneity were conducted using the R package ‘meta’ (version 4.9–2).

## Results

The studies included in this analysis are listed in Supplementary Table 1, available as Supplementary data at *IJE* online. The number of cases and controls with data for each risk factor varied, ranging from 23 755 cases and 30 153 controls with data for parity to 5078 cases and 6867 controls with data for cumulative lifetime intake of alcohol in the iCOGS dataset, and from 37 863 cases and 44 533 controls with data for parity to 12 213 cases and 13 232 controls with data for lifetime alcohol intake in the OncoArray dataset **(**Supplementary Tables 4 and 5, available as Supplementary data at *IJE* online).

The SNP associations with risk of overall as well as ER subtype breast cancer were consistent with those reported in literature^15^^,^^16^ (Supplementary Tables 2 and 3, available as Supplementary data at *IJE* online). The associations of the environmental risk factors with breast cancer risk were as expected in the population-based studies; in brief, age at menarche, being parous, number of full-term pregnancies, ever breastfeeding, cumulative duration of breastfeeding and premenopausal BMI were negatively associated with breast cancer risk, whereas age at first full-term pregnancy, ever use of oral contraceptives, postmenopausal BMI, current use of EPT, adult height, current smoking and cumulative alcohol consumption were all positively associated with breast cancer risk (Table 1; Supplementary Figures 1–3, available as Supplementary data at *IJE* online).

**Table 1.** Main effects for the epidemiological variables included in the analyses, derived from population-based studies only

Environmental risk factor	Overall breast cancer risk		ER-positive breast cancer risk		ER-negative breast cancer risk	
	Cases/controls	OR (95% CI)	Cases/controls	OR (95% CI)	Cases/controls	OR (95% CI)
Age at menarche (per 2 years)	36 893/46 854	0.91 (0.89-0.92)	26 630/46 854	0.91 (0.89-0.93)	4255/25 233	0.89 (0.85-0.93)
Ever parous (yes/no)	37 242/47 173	0.81 (0.77-0.84)	26 937/47 173	0.78 (0.74-0.81)	4309/25 585	0.94 (0.85-1.04)
Number of full-term pregnancies (1, 2, 3, ≥4)	31 390/41 215	0.87 (0.85-0.88)	22 720/41 215	0.86 (0.84-0.87)	3273/18 267	0.90 (0.86-0.94)
Age at first full-term pregnancy (per 5 years)^a^	30 168/39 850	1.14 (1.12-1.16)	21 869/39 850	1.17 (1.14-1.19)	3472/21 422	1.02 (0.97-1.06)
Ever breastfed (yes/no)^a^	27 786/30 582	0.91 (0.88-0.95)	19 691/30 582	0.92 (0.88-0.96)	3533/19 606	0.96 (0.88-1.03)
Duration of breastfeeding (per 12 months)^a^	24 553/25 524	0.96 (0.93-0.98)	17 355/25 524	0.95 (0.93-0.98)	3315/18 012	0.98 (0.94-1.03)
Adult height (per 5 cm)	35 767/46 506	1.09 (1.08-1.10)	25 763/46 506	1.10 (1.09-1.12)	3954/24 342	1.03 (1.00-1.05)
Premenopausal BMI (per 5 kg/m2)	7994/10 066	0.95 (0.92-0.98)	4835/9490	0.92 (0.89-0.95)	913/2030	1.07 (0.98-1.16)
Postmenopausal BMI (per 5 kg/m2)	27 495/32 495	1.07 (1.05-1.09)	20 503/32 283	1.07 (1.05-1.09)	1758/11 859	1.05 (1.00-1.11)
Ever use of oral contraceptives (yes/no)	35 126/44 608	1.22 (1.18-1.26)	25 271/44 608	1.24 (1.20-1.29)	3939/24 225	1.14 (1.05-1.23)
Current use of EPT (yes/no)^b,^^c^	16 637/17 946	1.75 (1.65-1.87)	12 566/17 946	1.93 (1.81-2.06)	1190/7353	1.11 (0.92-1.34)
Current use of ET (yes/no)^b,^^c^	16 444/17 920	1.10 (1.03-1.17)	11 829/16 844	1.11 (1.03-1.19)	936/6262	1.35 (1.11-1.64)
Lifetime intake of alcohol (per 10 g/day)	15 827/18 723	1.07 (1.05-1.10)	11 302/18 723	1.09 (1.07-1.11)	1612/11 562	1.03 (0.98-1.08)
Current smoking (yes/no)^d^	33 737/43 222	1.18 (1.13-1.24)	24 123/43 222	1.18 (1.12-1.25)	3707/22 573	1.06 (0.96-1.18)
Pack years smoked (per 10 pack-years)^e^	79 75/11 709	1.02 (1.00-1.04)	5944/11 709	1.02 (1.00-1.04)	896/6400	1.00 (0.95-1.04)

All models were adjusted for reference age and study.

a Among parous women.

b Among postmenopausal women.

c Additionally, models were adjusted for former use of menopausal hormonal therapy and use of any other menopausal hormonal therapy preparations.

d Additionally, model was adjusted for former smoking.

e Among ever smokers.

**Figure 1:**
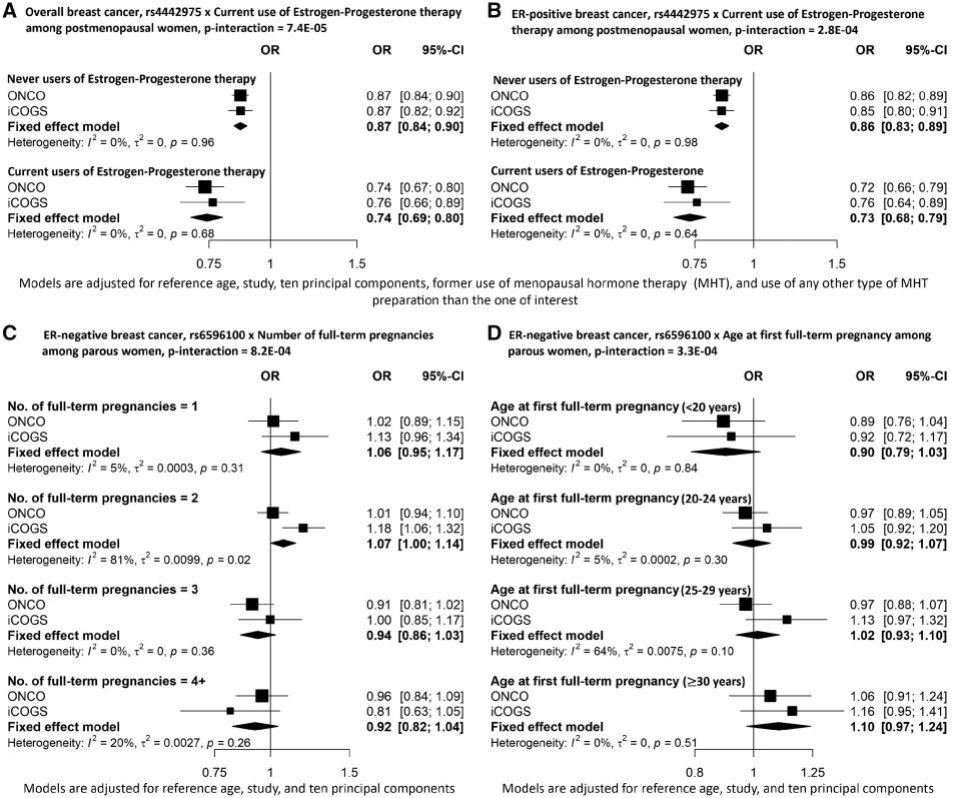
Odds ratios and 95% confidence intervals for associations between SNP and overall breast cancer (A), ER-positive breast cancer (B) and ER-negative breast cancer (C, D), stratified by categories of environmental risk factors.

We identified three SNP-risk factor interactions as noteworthy (BFDP <0.8) at ≤1% prior probability (Table 2). The strongest G x E interaction was found for SNP rs4442975 and current use of EPT [OR_meta-int_ = 0.85, 95% confidence interval (CI) = 0.78-0.92, *P*_meta-int_ = 7.4 x 10^–5^, BFDP = 0.73] with overall breast cancer at 0.1% prior probability. The minor allele of SNP rs4442975 was associated with a stronger reduced risk of breast cancer for current users of EPT (OR_meta_ = 0.74, 95% CI = 0.69-0.80) than for never users of MHT (OR_meta_ = 0.87, 95% CI = 0.84-0.90) (Figure 1A). This interaction was also found to be noteworthy at 1% prior probability for risk of ER-positive breast cancer (OR_meta-int_ = 0.85, 95% CI = 0.78-0.93, *P*_meta-int_ = 2.8 x 10^–4^, BFDP = 0.46). The association of rs4442975 with reduced risk of ER-positive breast cancer was stronger for current users of EPT (OR_meta_ = 0.73, 95% CI = 0.68-0.79) than for never MHT users (OR_meta_ = 0.86, 95% CI = 0.83-0.89) (Figure 1B).

**Table 2.** Gene-environment interactions with Bayesian false discovery probability (BFDP) <80% at ≤1% prior probability

		iCOGS	OncoArray	Meta-analysis		Prior probability (BFDP)					
Environmental risk factor	SNP (gene)	OR_int_**(95% CI)**	OR_int__**(95% CI)**_	OR_int_**(95% CI)**	p_int_	0.2	0.1	0.01	0.001	0.0001	ABF
**Overall breast cancer risk**											
Current EPT use^a^	rs4442975 (*IGFBP5*)	0.88 (0.75–1.03)	0.83 (0.76–0.92)	0.85 (0.78–0.92)	7.4E-05	0.011	0.023	0.209	0.727	0.964	0.003
**ER-positive breast cancer risk**											
Current EPT use^a^	rs4442975 (*IGFBP5*)	0.89 (0.75–1.06)	0.84 (0.75–0.93)	0.85 (0.78–0.93)	2.8E-04	0.033	0.072	0.462	0.896	0.989	0.009
**ER-negative breast cancer risk**											
Number of full-term pregnancies^b^^,^^c^	rs6596100 (*HSPA4*)	0.84 (0.75–0.93)	0.94 (0.87–1.01)	0.91 (0.85–0.96)	8.2E-04	0.104	0.207	0.742	0.967	0.997	0.029
Age at FFTP^b^	rs6596100 (*HSPA4*)	1.13 (1.02–1.26)	1.11 (1.03–1.19)	1.12 (1.05–1.19)	3.3E-04	0.048	0.103	0.558	0.927	0.992	0.012

ER: Estrogen receptor, OR_int_: Interaction odds ratio, CI: Confidence interval, SNP: Single nucleotide polymorphism, ABF: Approximate Bayes Factor, EPT: Estrogen-Progesterone therapy, FFTP: First full-term pregnancy.

a Among postmenopausal women only.

b Among parous women only.

c Categories: 1, 2, 3, ≥4.

The two other noteworthy SNP-risk factor interactions were found for ER-negative breast cancer risk. The interaction between rs6596100 and number of full-term pregnancies was noteworthy at 1% prior probability (OR_meta-int_ = 0.91, 95% CI = 0.85-0.96, *P*_meta-int_ = 8.2 x 10^–4^, BFDP = 0.74). The minor allele of the rs6596100 variant was associated with a reduced risk of overall breast cancer (OR_meta_ = 0.96, 95% CI = 0.94-0.98) and ER-positive breast cancer (OR_meta_ = 0.94, 95% CI = 0.92-0.96), respectively, but not ER-negative breast cancer (OR_meta_ = 1.01, 95% CI = 0.97-1.05). The rs6596100 associated risk of ER-negative breast cancer appears to decrease with number of full-term pregnancies for parous women, with the estimated per-allele OR_meta_ being 1.06 (95% CI = 0.95-1.17) for women who had had one full-term pregnancy and 0.92 (95% CI = 0.82-1.04) for women who had had four or more full-term pregnancies (Figure 1C).

For parous women, we observed noteworthy evidence that the ER-negative breast cancer risk associated with rs6596100 was also modified by age at first full-term pregnancy (OR_meta-int_ = 1.12, 95% CI = 1.05-1.19, *P*_meta-int_ = 3.3 x 10^–4^, BFDP = 0.56). The risk conferred by rs6596100 on ER-negative breast cancer was decreased for women with age at first full-term pregnancy below 20 years (OR_meta_ = 0.90, 95% CI = 0.79-1.03) but increased for women with age at first full-term pregnancy ≥30 years (OR_meta_ = 1.10, 95% CI = 0.97-1.24) (Figure 1D). However, we observed between-study heterogeneity for the interaction between rs6596100 and age at first full-term pregnancy (Supplementary Figure 4, available as Supplementary data at *IJE* online). Several other interactions were found to be noteworthy (BFDP <0.8) at 5% prior probability (Supplementary Table 6, available as Supplementary data at *IJE* online). Meta-analysed results of all the G x E interactions for overall and ER subtype risk are shown in Supplementary Tables 7–9, available as Supplementary data at *IJE* online.

In replication analyses, we found evidence for two previously reported associations in the independent subset of OncoArray data (Supplementary Table 10, available as Supplementary data at *IJE* online). We estimated an interaction OR for overall breast cancer of 0.80 (95% CI = 0.69-0.93, *P*_int_ = 0.004) for current EPT use and rs13387042, a SNP for which we had previously reported an interaction OR of 0.83 (95% CI = 0.74-0.94, *P*_int_ = 2.43 x 10^–3^).^20^ SNP rs13387042 is in strong linkage disequilibrium with rs4442975; hence this result is consistent with the interaction observed for rs4442975 in the full dataset. In addition, we also observed evidence for a G x E interaction between rs941764 and cumulative lifetime intake of alcohol (<20 g/day vs ≥20 g/day) with ER-negative breast cancer risk (OR_int_ = 0.64, 95% CI = 0.45-0.92, *P*_int_ = 0.01), compared with OR_int_ of 0.53 (95% CI = 0.36-0.76, *P*_int_ = 6.8 x 10^–4^) in Rudolph *et al.*^54^ The corresponding meta-analysed interaction OR (per 10 g/day cumulative lifetime alcohol intake) based on OncoArray and iCOGS datasets was 0.90 (95% CI = 0.81-0.99, *P*_int_ = 0.03). For the G x E interaction between SNP rs3817198 and number of children for parous women, which had the strongest evidence for overall risk of breast cancer in previous analyses (OR_int_ = 1.06, 95% CI =1.04-1.08, *P*_int_ = 2.4 x 10^–06^),^20^ there was weak evidence of interaction, but in the opposite direction in the replication analyses (OR_int_ = 0.94, 95% CI = 0.94-1.00, *P*_int_ = 0.03).

## Discussion

In this study, we evaluated all known common susceptibility loci for interactions with breast cancer risk factors, and found little evidence for departures from a multiplicative model. We refer to G x E interactions as effect modification conferred by epidemiological risk factors on the association between SNPs and breast cancer risk, but it can very well be SNPs modifying the association of risk factors with breast cancer risk. We identified three noteworthy (BFDP <0.8) G x E interactions related to breast cancer risk based on prior probabilities ≤1%. The strongest evidence was found for effect modification between rs4442975 and current use of EPT with overall and ER-positive breast cancer risk. Moreover, we found evidence of interactions between the SNP rs6596100 and number of full-term pregnancies and age at first full-term pregnancy, respectively, for ER-negative breast cancer risk.

The SNP rs4442975 is located in an intergenic region on the long arm of chromosome 2 (2q35). Another SNP within the same genomic region, rs13387042, was previously reported to show an interaction also with current use of EPT.^20^ We replicated this interaction between rs13387042 and current use of EPT using the OncoArray dataset. The two SNPs rs13387042 and rs4442975 are highly correlated (r^2^ = 0.93) and conditional analysis yielded a significant association only for rs4442975, so that these results reflect the same interaction. Fine-mapping and functional analyses have identified rs4442975 to be the most likely causal variant in this region.^43^ Thus despite the small difference in the risk estimates between never and current EPT, replication of this G x E interaction reinforced what we found previously, implicating the role of the *IGFBP5* gene and estrogen pathway in breast cancer.

Functional analyses indicate that SNP rs4442975 lies near a transcriptional enhancer which physically interacts with the *IGFBP5* promoter, suggesting that the T-allele of rs4442975 decreases susceptibility to breast cancer via increased expression of insulin-like growth factor binding protein 5 (IGFBP5).^43^ IGFBP5 is a key member of the insulin-like growth factor (IGF) axis which plays an important role in cellular differentiation, proliferation and apoptosis in breast cancer.^55^ Activation of the IGF receptors by IGF causes phosphorylation of insulin receptor substrates (IRS-1 and IRS-2). This phosphorylation cascades multiple downstream signalling pathways such as Ras/mitogen-activated protein kinase (MAPK) and phosphoinositide (PI3K) serine-threonine kinase (AkT), which play a role in breast carcinogenesis.^56^^,^^57^ Estrogen can stimulate the IGF pathway via increased expression of both insulin-like growth factor receptor-1 and IRS-1. Some studies have also reported a positive correlation between overexpression of IGFBP5 and the presence of ER in breast cancer cell lines. Progesterone has been shown to act by increasing levels of IRS-2 and sensitizing breast cancer cells to downstream signalling pathways such as MAPK and Akt.^58–60^ It is plausible that exogenous hormone exposure due to estrogen and progesterone therapy may affect the regulation of the IGF pathway and thereby modulate germline *IGFPB5* variant-related susceptibility to breast cancer. Note however that two other independent breast cancer risk variants in this region (tagged by rs16857609^13^ and a 1.3 kb insertion/deletion^49^) are also believed to target *IGFBP5*, but we did not find evidence for interactions between these variants and current EPT use.

Women of young age at first pregnancy are known to have increased circulating sex hormone-binding globulin and prolactin but decreased total estrogen levels.^61^^,^^62^ Likewise, women who have had multiple full-term pregnancies have an overall decreased lifetime exposure to estrogen.^61^^,^^63^^,^^64^ The association of rs6596100 with ER-negative breast cancer risk was found to be modified by number of full-term pregnancies and age at first full-term pregnancy for parous women. Based on INQUISIT,^15^ the target genes of rs6596100 and highly correlated SNPs are predicted to be heat shock protein family A member 4 (*HSPA4*) and AF4/FMR2 family member 4 (*AFF4)*. INQUISIT predicts *HSPA4* as the most likely target, due to overlap of multiple correlated SNPs lying in *HSPA4* promoter region, distal regulatory elements and coding sequence. *HSPA4* gene is responsible for production of heat shock proteins (Hsps), particularly those belonging to the family HSP70. The underlying mechanisms regarding the relationship between rs6596100 and these pregnancy-related risk factors are unknown at present. It is plausible that a lower estrogenic milieu due to reproductive factors may affect the formation of multicomplexes between steroid receptors like ER and heat shock proteins (HSPs), therefore affecting signalling pathways such as Wnt, ErbB, serine/threonine and tyrosine protein kinase, which are known to be involved in breast carcinogenesis. Whereas there is some biological plausibility regarding the observed interactions with rs6596100, the findings nevertheless could be by chance, and thus require independent replication.

The SNP rs941764 is located on chromosome 14 in intron of *CCDC88C* gene.^15^^,^^22^ The effect modification of rs941764-associated ER-negative breast cancer risk by lifetime intake of alcohol was first reported by Rudolph *et al.*^54^ We replicated this G x E interaction in an independent dataset in our study. Mutations in this gene region have been associated with dysregulation of Wnt signalling in neural disorders such as congenital hydrocephalus.^65^ This gene codes a Hook-related protein (HkRP2) that binds to an important scaffold protein, Dishevelled, in the Wnt signalling pathway, affecting all downstream activity.^65^

A role of alcohol has been well recognized in initiation and progression of breast cancer, presumably via multiple cellular and molecular mechanisms, including the EGFR/ErbB2 pathways. Downstream to EGFR/ErbB2 pathways lie multiple pathways such as the MAPK, Wnt/GSK3β/β-catenin pathways.^66^ Therefore, alcohol consumption could affect the risk of ER-negative breast cancer through dysregulation of Wnt signalling.

Our study provides the most comprehensive evaluation to date of potential effect modification of all known common genetic susceptibility variants by environmental risk factors for breast cancer. Our findings are based on the largest available dataset on breast cancer. Despite its large sample size, the study may remain statistically underpowered, considering the rather modest effect sizes of most of the common variants associated with breast cancer risk, and particularly for risk factors for which we have fewer data (Supplementary Table 11, available as Supplementary data at *IJE* online).^18^ Statistical power was further diminished for subtype-specific analyses due to reduced sample sizes, especially for ER-negative breast cancer (10 896 ER-negative cases in the combined iCOGS and OncoArray dataset).^18^ The lack of strong effect modifications for breast cancer could also be explained by the overall weak to moderate associations of environmental risk factors, except for MHT use with breast cancer risk along with the modest associations of common genetic variants. A further limitation of our study is that the findings may not be generalizable to other racial/ethnic groups since the analyses were restricted to women of European ancestry.

In conclusion, our analyses suggest that most of the associated effects of breast cancer susceptibility loci and environmental risk factors are consistent with a multiplicative model. The strongest evidence for an interaction was between the candidate causal variant rs4442975 at 2q35 and current use of EPT. The associated effect is supported by a plausible underlying biological mechanism, but further epidemiological and functional validation will be required to determine whether the interaction is genuine. The newly reported results for ER-negative breast cancer risk generate plausible biological hypotheses and may inform future functional studies. Overall, the results from our analyses do not suggest strong effect modification of the association between breast cancer susceptibility loci and risk of breast cancer by established epidemiological risk factors.

## Funding

This work was supported by the following funding agencies. The Breast Cancer Association Consortium is funded by Cancer Research UK [C1287/A16563, C1287/A10118], the European Union's Horizon 2020 Research and Innovation Programme (grant numbers 634935 and 633784 for BRIDGES and B-CAST respectively) and by the European Communitýs Seventh Framework Programme under grant agreement number 223175 (grant number HEALTH-F2-2009–223175) (COGS). The EU Horizon 2020 Research and Innovation Programme funding source had no role in study design, data collection, data analysis, data interpretation or writing of the report.

Genotyping of the OncoArray was funded by the NIH Grant U19 CA148065 and Grant C1287/A16563 and the PERSPECTIVE project supported by the Government of Canada through Genome Canada and the Canadian Institutes of Health Research (grant GPH-129344) and the Ministère de l’Économie, Science et Innovation du Québec through Genome Québec and the PSRSIIRI-701 grant and the Quebec Breast Cancer Foundation. Funding for the iCOGS infrastructure came from: the European Community's Seventh Framework Programme under grant agreement n° 223175 (HEALTH-F2-2009–223175) (COGS), Cancer Research UK (C1287/A10118, C1287/A10710, C12292/A11174, C1281/A12014, C5047/A8384, C5047/A15007, C5047/A10692, C8197/A16565), the National Institutes of Health (CA128978) and Post-Cancer GWAS initiative (1U19 CA148537, 1U19 CA148065 and 1U19 CA148112: the GAME-ON initiative), the Department of Defence (W81XWH-10–1-0341), the Canadian Institutes of Health Research (CIHR) for the CIHR Team in Familial Risks of Breast Cancer, and Komen Foundation for the Cure, the Breast Cancer Research Foundation and the Ovarian Cancer Research Fund. The DRIVE Consortium was funded by U19 CA148065.

The Australian Breast Cancer Family Study (ABCFS) was supported by grant UM1 CA164920 from the National Cancer Institute (USA). The content of this manuscript does not necessarily reflect the views or policies of the National Cancer Institute or any of the collaborating centers in the Breast Cancer Family Registry (BCFR), nor does mention of trade names, commercial products or organizations imply endorsement by the USA Government or the BCFR. The ABCFS was also supported by the National Health and Medical Research Council of Australia, the New South Wales Cancer Council, the Victorian Health Promotion Foundation (Australia) and the Victorian Breast Cancer Research Consortium. J.L.H. is a National Health and Medical Research Council (NHMRC) Senior Principal Research Fellow. M.C.S. is an NHMRC Senior Research Fellow. The ABCS study was supported by the Dutch Cancer Society [grants NKI 2007–3839; 2009 4363]. The Australian Breast Cancer Tissue Bank (ABCTB) is generously supported by the National Health and Medical Research Council of Australia, the Cancer Institute NSW and the National Breast Cancer Foundation. The AHS study is supported by the intramural research programme of the National Institutes of Health, the National Cancer Institute (grant number Z01-CP010119) and the National Institute of Environmental Health Sciences (grant number Z01-ES049030). The work of the BBCC was partly funded by ELAN-Fond of the University Hospital of Erlangen. The BCEES was funded by the National Health and Medical Research Council, Australia and the Cancer Council Western Australia and acknowledges funding from the National Breast Cancer Foundation (JS). The BREast Oncology GAlician Network (BREOGAN) is funded by Acción Estratégica de Salud del Instituto de Salud Carlos III FIS PI12/02125/Cofinanciado FEDER; Acción Estratégica de Salud del Instituto de Salud Carlos III FIS Intrasalud (PI13/01136); Programa Grupos Emergentes, Cancer Genetics Unit, Instituto de Investigacion Biomedica Galicia Sur, Xerencia de Xestion Integrada de Vigo-SERGAS, Instituto de Salud Carlos III, Spain; Grant 10CSA012E, Consellería de Industria Programa Sectorial de Investigación Aplicada, PEME I + D e I + D Suma del Plan Gallego de Investigación, Desarrollo e Innovación Tecnológica de la Consellería de Industria de la Xunta de Galicia, Spain; Grant EC11-192, Fomento de la Investigación Clínica Independiente, Ministerio de Sanidad, Servicios Sociales e Igualdad, Spain; and Grant FEDER-Innterconecta. Ministerio de Economia y Competitividad, Xunta de Galicia, Spain. CBCS is funded by the Canadian Cancer Society (grant # 313404) and the Canadian Institutes of Health Research. The CECILE study was supported by Fondation de France, Institut National du Cancer (INCa), Ligue Nationale contre le Cancer, Agence Nationale de Sécurité Sanitaire, de l'Alimentation, de l'Environnement et du Travail (ANSES), Agence Nationale de la Recherche (ANR). The CGPS was supported by the Chief Physician Johan Boserup and Lise Boserup Fund, the Danish Medical Research Council and Herlev and Gentofte Hospital. The CNIO-BCS was supported by the Instituto de Salud Carlos III, the Red Temática de Investigación Cooperativa en Cáncer and grants from the Asociación Española Contra el Cáncer and the Fondo de Investigación Sanitario (PI11/00923 and PI12/00070). The American Cancer Society funds the creation, maintenance, and updating of the CPS-II cohort. The CTS was supported by the California Breast Cancer Act of 1993, the California Breast Cancer Research Fund (contract 97–10500) and the National Institutes of Health (R01 CA77398, K05 CA136967, UM1 CA164917, and U01 CA199277). Collection of cancer incidence data was supported by the California Department of Public Health as part of the statewide cancer reporting programme mandated by California Health and Safety Code Section 103885. H.A.C .receives support from the Lon V Smith Foundation (LVS39420). The coordination of EPIC is financially supported by the European Commission (DG-SANCO) and the International Agency for Research on Cancer. The national cohorts are supported by: Ligue Contre le Cancer, Institut Gustave Roussy, Mutuelle Générale de l’Education Nationale, Institut National de la Santé et de la Recherche Médicale (INSERM) (France); German Cancer Aid, German Cancer Research Center (DKFZ), Federal Ministry of Education and Research (BMBF) (Germany); the Hellenic Health Foundation, the Stavros Niarchos Foundation (Greece); Associazione Italiana per la Ricerca sul Cancro-AIRC-Italy and National Research Council (Italy); Dutch Ministry of Public Health, Welfare and Sports (VWS), Netherlands Cancer Registry (NKR), LK Research Funds, Dutch Prevention Funds, Dutch ZON (Zorg Onderzoek Nederland), World Cancer Research Fund (WCRF), Statistics Netherlands (The Netherlands); Health Research Fund (FIS), PI13/00061 to Granada, PI13/01162 to EPIC-Murcia, Regional Governments of Andalucía, Asturias, Basque Country, Murcia and Navarra, ISCIII RETIC (RD06/0020) (Spain); Cancer Research UK (14136 to EPIC-Norfolk; C570/A16491 and C8221/A19170 to EPIC-Oxford), Medical Research Council (1000143 to EPIC-Norfolk, MR/M012190/1 to EPIC-Oxford) (United Kingdom). The ESTHER study was supported by a grant from the Baden Württemberg Ministry of Science, Research and Arts. Additional cases were recruited in the context of the VERDI study, which was supported by a grant from the German Cancer Aid (Deutsche Krebshilfe). The GENICA was funded by the Federal Ministry of Education and Research (BMBF) Germany grants 01KW9975/5, 01KW9976/8, 01KW9977/0 and 01KW0114, the Robert Bosch Foundation, Stuttgart, Deutsches Krebsforschungszentrum (DKFZ), Heidelberg, the Institute for Prevention and Occupational Medicine of the German Social Accident Insurance, Institute of the Ruhr University Bochum (IPA), Bochum, as well as the Department of Internal Medicine, Evangelische Kliniken Bonn gGmbH, Johanniter Krankenhaus, Bonn, Germany. The GESBC was supported by the Deutsche Krebshilfe e. V. [70492] and the German Cancer Research Center (DKFZ). The KARMA study was supported by Märit and Hans Rausings Initiative Against Breast Cancer. kConFab is supported by a grant from the National Breast Cancer Foundation, and previously by the National Health and Medical Research Council (NHMRC), the Queensland Cancer Fund, the Cancer Councils of New South Wales, Victoria, Tasmania and South Australia, and the Cancer Foundation of Western Australia. Financial support for the AOCS was provided by the United States Army Medical Research and Materiel Command [DAMD17-01–1-0729], Cancer Council Victoria, Queensland Cancer Fund, Cancer Council New South Wales, Cancer Council South Australia, Cancer Foundation of Western Australia, Cancer Council Tasmania and the National Health and Medical Research Council of Australia (NHMRC; 400413, 400281, 199600). G.C.T. and P.W. are supported by the NHMRC. R.B. was a Cancer Institute NSW Clinical Research Fellow. L.M.B.C. is supported by the ‘Stichting tegen Kanker’. D.L. is supported by the FWO. The MARIE study was supported by the Deutsche Krebshilfe e.V. [70–2892-BR I, 106332, 108253, 108419, 110826, 110828], the Hamburg Cancer Society, the German Cancer Research Center (DKFZ) and the Federal Ministry of Education and Research (BMBF) Germany [01KH0402]. The MCBCS was supported by the NIH grants CA192393, CA116167, CA176785 an NIH Specialized Program of Research Excellence (SPORE) in Breast Cancer [CA116201] and the Breast Cancer Research Foundation and a generous gift from the David F. and Margaret T. Grohne Family Foundation. MCCS cohort recruitment was funded by VicHealth and Cancer Council Victoria. The MCCS was further supported by Australian NHMRC grants 209057 and 396414, and by infrastructure provided by Cancer Council Victoria. Cases and their vital status were ascertained through the Victorian Cancer Registry (VCR) and the Australian Institute of Health and Welfare (AIHW), including the National Death Index and the Australian Cancer Database. The MEC was support by NIH grants CA63464, CA54281, CA098758, CA132839 and CA164973. The MISS study is supported by funding from ERC-2011–294576 Advanced grant, Swedish Cancer Society, Swedish Research Council, local hospital funds, Berta Kamprad Foundation, Gunnar Nilsson. The MMHS study was supported by NIH grants CA97396, CA128931, CA116201, CA140286 and CA177150. M.S.K.C.C. is supported by grants from the Breast Cancer Research Foundation and Robert and Kate Niehaus Clinical Cancer Genetics Initiative. The NBHS was supported by NIH grant R01CA100374. Biological sample preparation was conducted by the Survey and Biospecimen Shared Resource, which is supported by P30 CA68485. The Northern California Breast Cancer Family Registry (NC-BCFR) and Ontario Familial Breast Cancer Registry (OFBCR) were supported by grant UM1 CA164920 from the National Cancer Institute (USA). The content of this manuscript does not necessarily reflect the views or policies of the National Cancer Institute or any of the collaborating centers in the Breast Cancer Family Registry (BCFR), nor does mention of trade names, commercial products, or organizations imply endorsement by the USA Government or the BCFR. The Carolina Breast Cancer Study was funded by Komen Foundation, the National Cancer Institute (P50 CA058223, U54 CA156733, U01 CA179715) and the North Carolina University Cancer Research Fund. The NHS was supported by NIH grants P01 CA87969, UM1 CA186107 and U19 CA148065. The NHS2 was supported by NIH grants UM1 CA176726 and U19 CA148065. The PBCS was funded by Intramural Research Funds of the National Cancer Institute, Department of Health and Human Services, USA. Genotyping for PLCO was supported by the Intramural Research Program of the National Institutes of Health, NCI, Division of Cancer Epidemiology and Genetics. The PLCO is supported by the Intramural Research Program of the Division of Cancer Epidemiology and Genetics and supported by contracts from the Division of Cancer Prevention, National Cancer Institute, National Institutes of Health. PROCAS is funded from NIHR grant PGfAR 0707–10031. D.G.E. is supported by the NIHR Biomedical Research Centre in Manchester (IS-BRC-1215–20007). The SASBAC study was supported by funding from the Agency for Science, Technology and Research of Singapore (A*STAR), the US National Institute of Health (NIH) and the Susan G. Komen Breast Cancer Foundation. The SBCS was supported by Sheffield Experimental Cancer Medicine Centre and Breast Cancer Now Tissue Bank. SEARCH is funded by Cancer Research UK [C490/A10124, C490/A16561] and supported by the UK National Institute for Health Research Biomedical Research Centre at the University of Cambridge. The University of Cambridge has received salary support for P.D.P.P. from the NHS in the East of England through the Clinical Academic Reserve. The Sister Study (SISTER) is supported by the Intramural Research Program of the NIH, National Institute of Environmental Health Sciences (Z01-ES044005 and Z01-ES049033). The SMC is funded by the Swedish Cancer Foundation. The UCIBCS component of this research was supported by the NIH [CA58860, CA92044] and the Lon V Smith Foundation [LVS39420]. The UKBGS is funded by Breast Cancer Now and the Institute of Cancer Research (ICR), London. ICR acknowledges NHS funding to the NIHR Biomedical Research Centre. The USRT Study was funded by Intramural Research Funds of the National Cancer Institute, Department of Health and Human Services, USA. The WHI programme is funded by the National Heart, Lung, and Blood Institute, the US National Institutes of Health and the US Department of Health and Human Services (HHSN268201100046C, HHSN268201100001C, HHSN268201100002C, HHSN268201100003C, HHSN268201100004C and HHSN271201100004C). This work was also funded by NCI U19 CA148065-01.

## Supplementary Material

dyz193_Supplementary_Data
