# Supplementary material for: Assessment of interactions between 205 breast cancer susceptibility loci and 13 established risk factors in relation to breast cancer risk, in the Breast Cancer Association Consortium
Source: Int J Epidemiol. 2019 Oct 12;49(1):216–32. doi: 10.1093/ije/dyz193 (PMC7426027; doi:10.1093/ije/dyz193)

**Supplementary Figure 1.** Forest plot of meta-analyzed **study-wise** odds ratios and 95% confidence intervals of population-based studies for associations between environmental risk factors and **overall breast cancer risk**: (A) age at menarche (per 2 years), (B) Parity (yes/no), (C) number of full-term pregnancies (among parous, per pregnancy), (D) ever breastfed (no/yes), (E) duration of breastfeeding (among parous, per 12 months), (F) age at first full-term pregnancy (per 5 years), (G) adult BMI, premenopausal women (per 5 kg/m^2^), (H) adult BMI, postmenopausal women (per 5 kg/m^2^), (I) adult height (per 5 cm), (J) ever use of oral contraceptives (yes/no), (K) current use of combined estrogen-progesterone therapy (yes/no), (L) current use of estrogen-only therapy (yes/no), (M) lifetime intake of alcohol (per 10 g/day), (N) current smoking (yes/no), (O) smoking amount (per 10 pack-years)

1. Age at menarche (per 2 years)


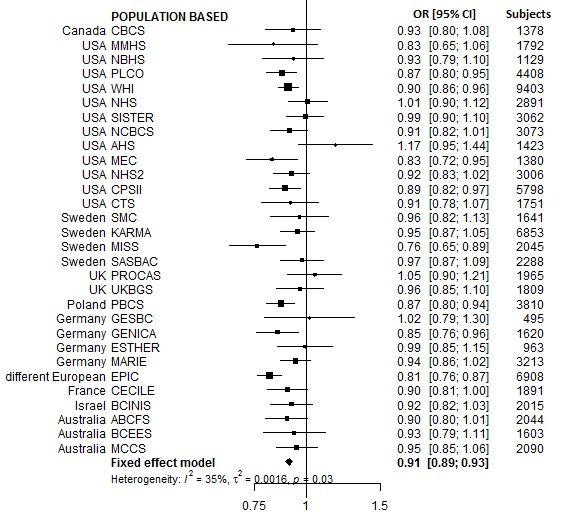


1. Parity (yes/no)


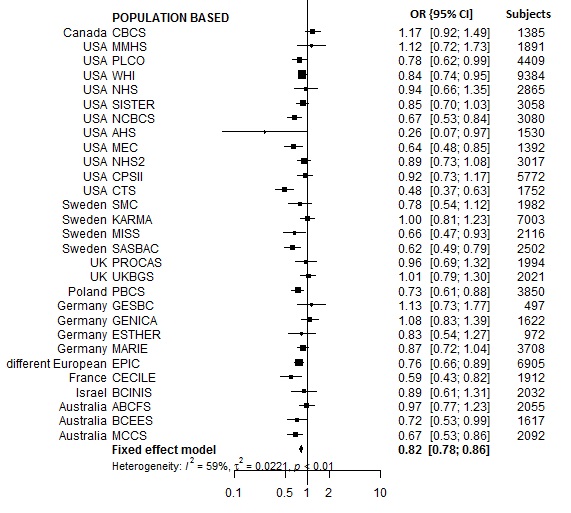


1. Number of full-term pregnancies (per pregnancy) among parous women


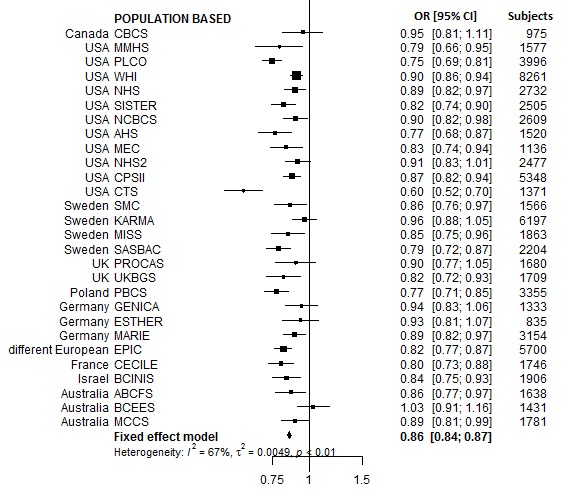


1. Ever breastfed (yes/no) among parous women


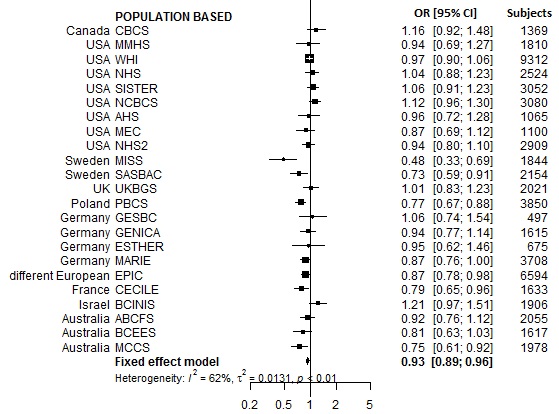


1. Duration of breastfeeding (per 12 months) among parous women


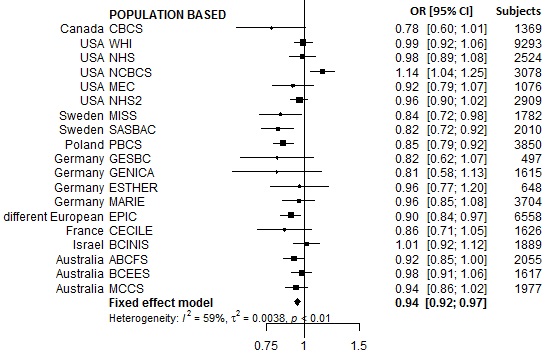


1. Age at first full-term pregnancy (per 5 years) among parous women


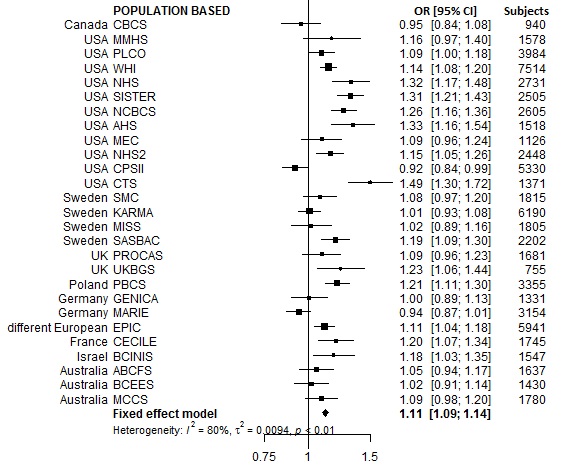


1. Adult BMI, premenopausal women (per 5 kg/m^2^)


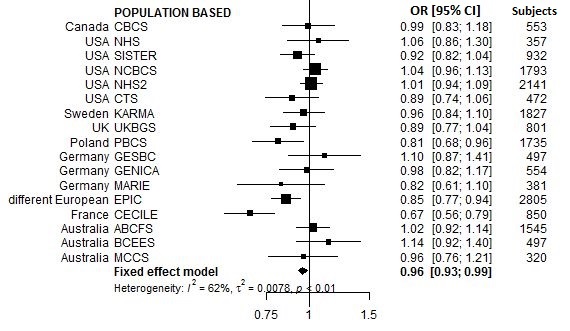


1. Adult BMI, postmenopausal women (per 5 kg/m^2^)


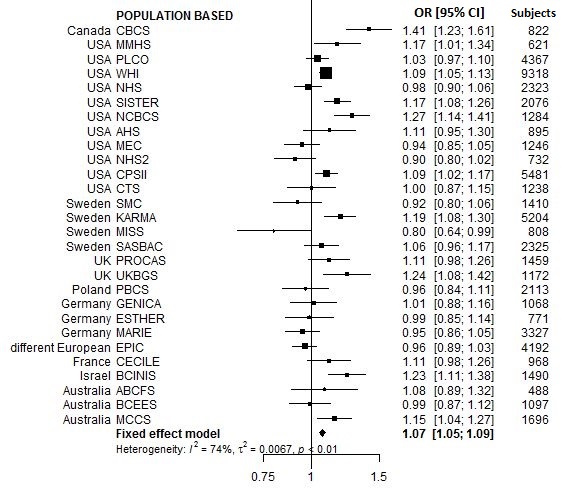


1. Adult height (per 5 cm)


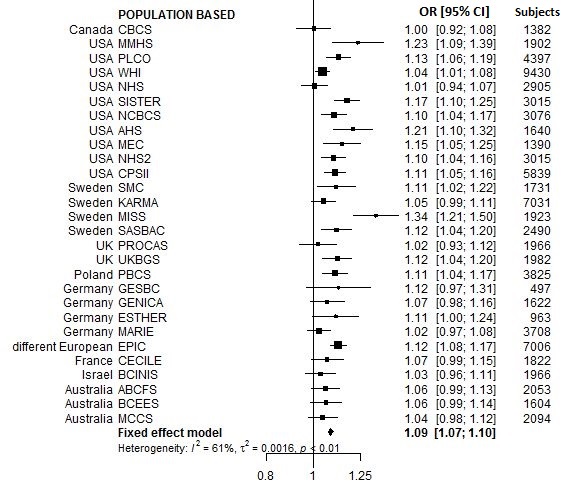


1. Ever use of oral contraceptives


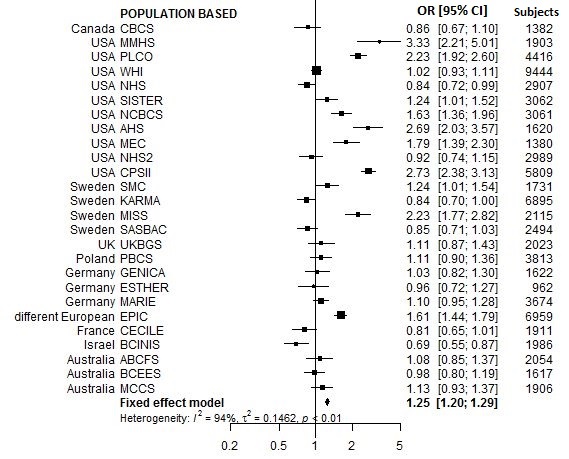


1. Current use of combined estrogen-progesterone therapy (among postmenopausal women, yes/no)


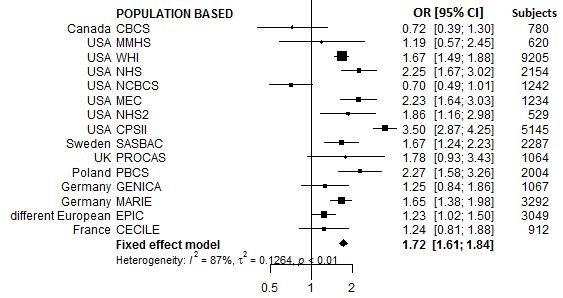


1. Current use of estrogen-only therapy (among postmenopausal women, yes/no)


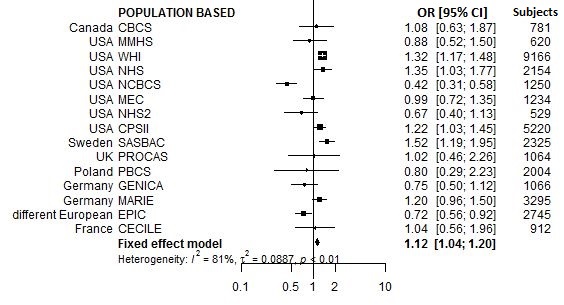


1. Lifetime intake of alcohol (per 10g/day)


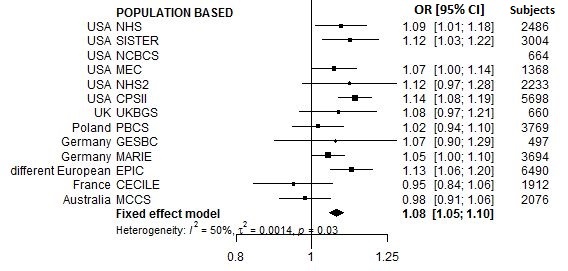


1. Current smoking (yes/no)


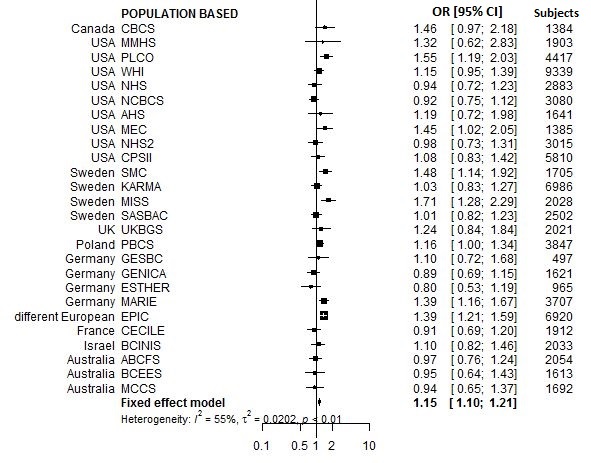


1. Smoking amount (per 10 pack-years)


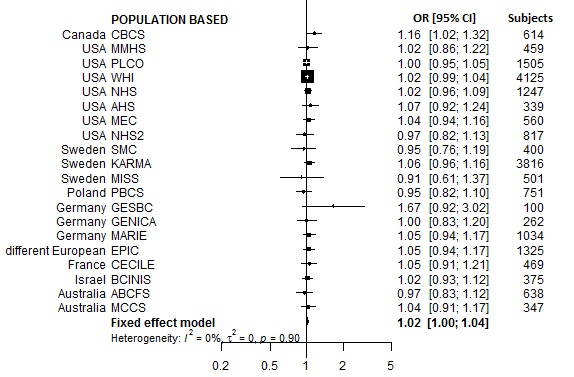


**Supplementary Figure 2.** Forest plot of meta-analyzed **study-wise** odds ratios and 95% confidence intervals of population-based studies for associations between environmental risk factors and **ER-positive breast cancer risk**: (A) age at menarche (per 2 years), (B) Parity (yes/no), (C) number of full-term pregnancies (among parous, per pregnancy), (D) ever breastfed (no/yes), (E) duration of breastfeeding (among parous, per 12 months), (F) age at first full-term pregnancy (per 5 years), (G) adult BMI, premenopausal women (per 5 kg/m^2^), (H) adult BMI, postmenopausal women2 (per 5 kg/m^2^), (I) adult height (per 5 cm), (J) ever use of oral contraceptives (yes/no), (K) current use of combined estrogen-progesterone therapy (yes/no), (L) current use of estrogen-only therapy (yes/no), (M) lifetime intake of alcohol (per 10 g/day), (N) current smoking (yes/no), (O) smoking amount (per 10 pack-years)

1. Age at menarche (per 2 years)


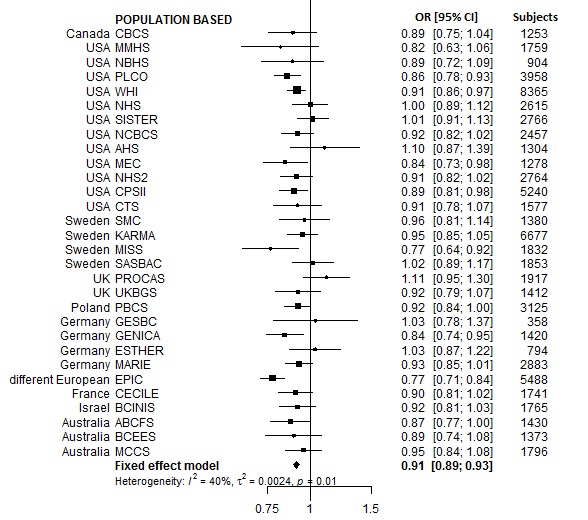


1. Parity (yes/no)


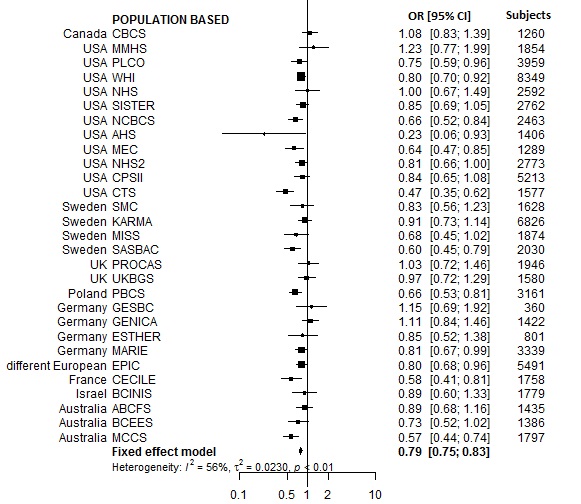


1. Number of full-term pregnancies (per pregnancy) among parous women


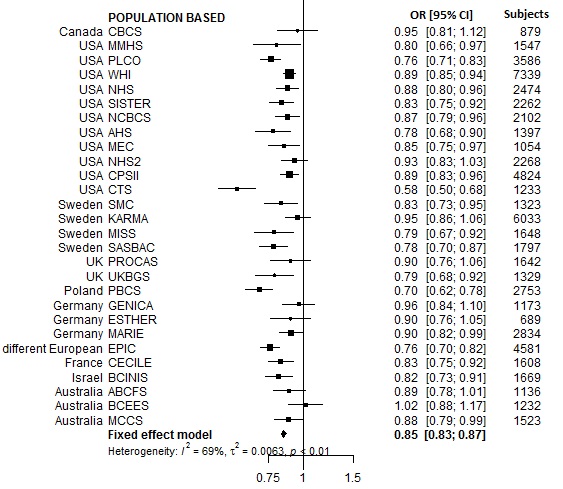


1. Ever breastfed (yes/no) among parous women


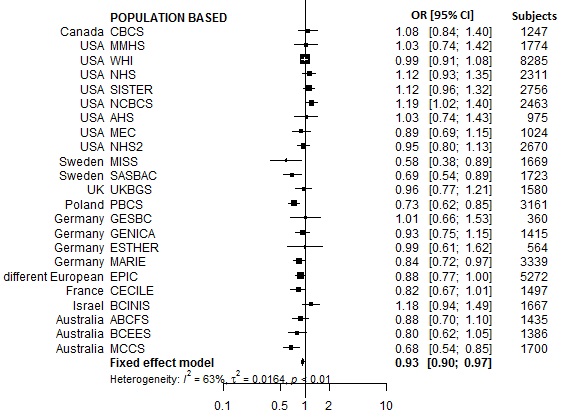


1. Duration of breastfeeding (per 12 months) among parous women


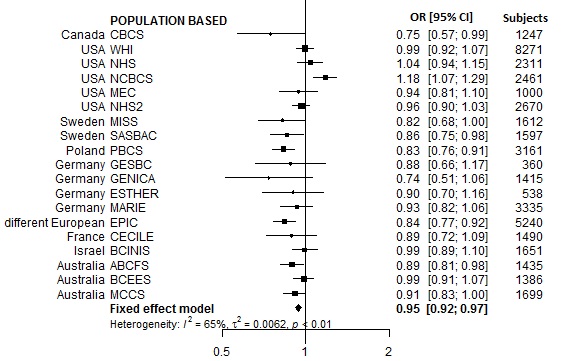


1. Age at first full-term pregnancy (per 5 years) among parous women


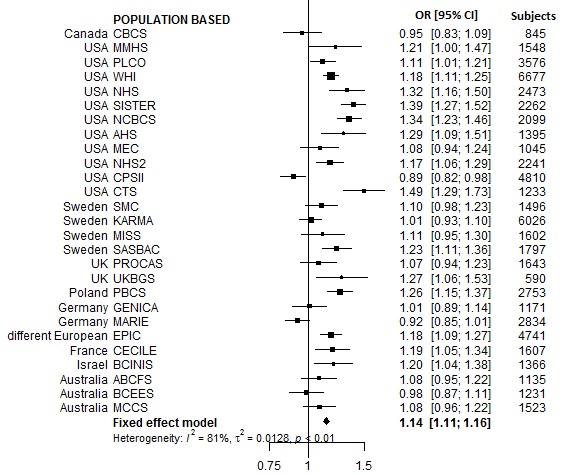


1. Adult BMI, premenopausal women (per 5 kg/m^2^)


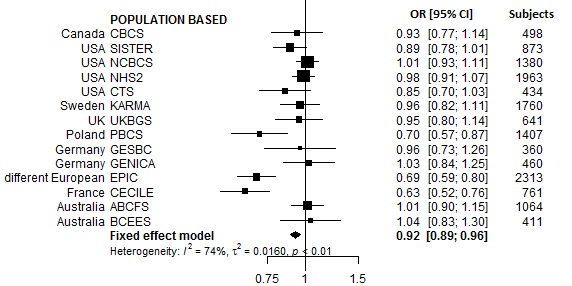


1. Adult BMI, postmenopausal women (per 5 kg/m^2^)


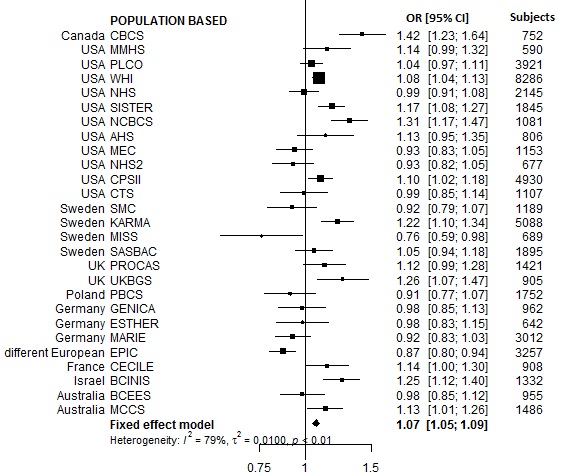


1. Adult height (per 5 cm)


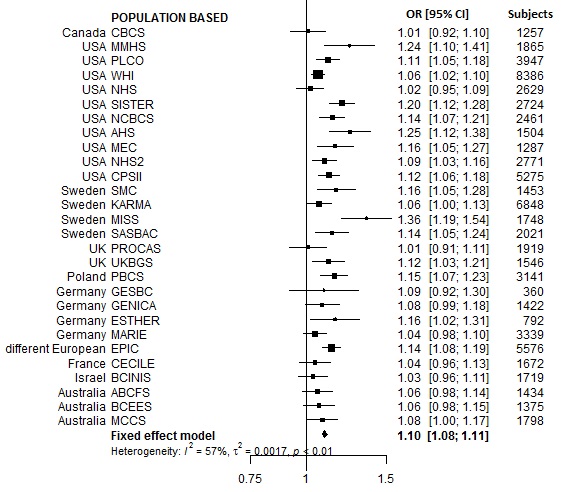


1. Ever use of oral contraceptives


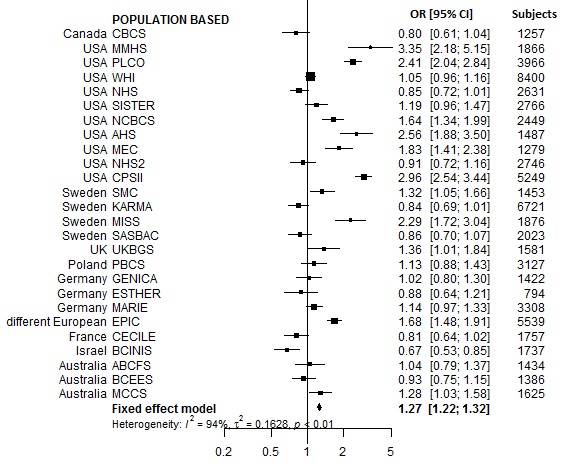


1. Current use of combined estrogen-progesterone therapy (among postmenopausal women, yes/no)


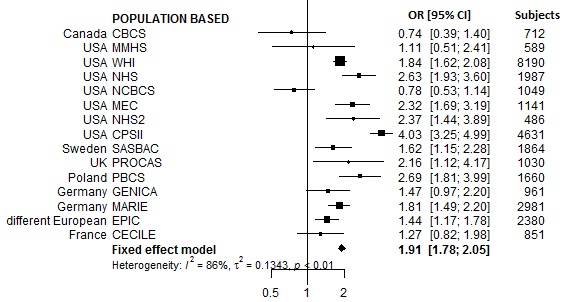


1. Current use of estrogen-only therapy (among postmenopausal women, yes/no)


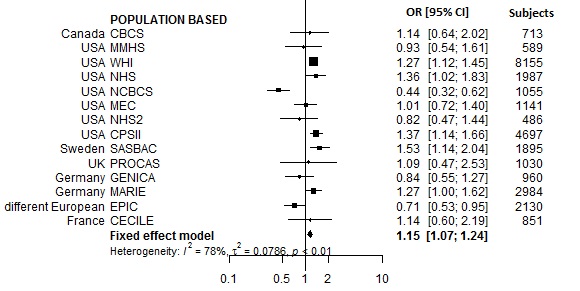


1. Lifetime intake of alcohol (per 10g/day)


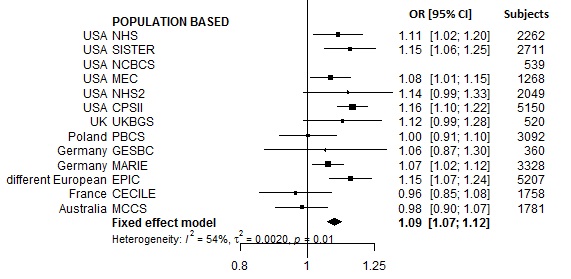


1. Current smoking (yes/no)


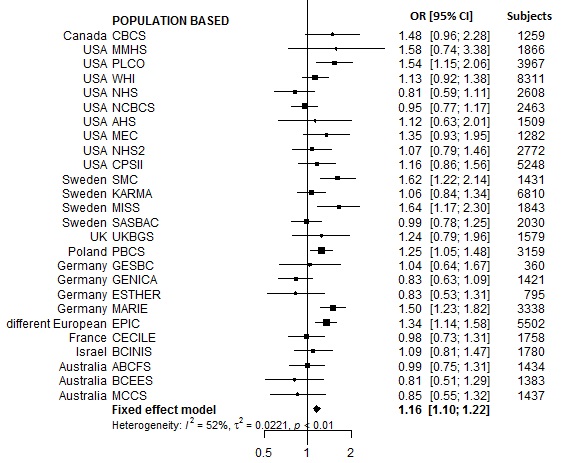


1. Smoking amount (per 10 pack-years)


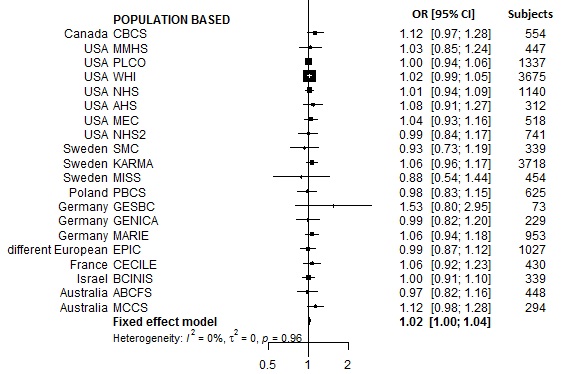


**Supplementary Figure 3.** Forest plot of meta-analyzed **study-wise** odds ratios and 95% confidence intervals of population-based studies for associations between environmental risk factors and **ER-negative breast cancer risk**: (A) age at menarche (per 2 years), (B) Parity (yes/no), (C) number of full-term pregnancies (among parous, per pregnancy), (D) ever breastfed (no/yes), (E) duration of breastfeeding (among parous, per 12 months), (F) age at first full-term pregnancy (per 5 years), (G) adult BMI, premenopausal women (per 5 kg/m^2^), (H) adult BMI, postmenopausal women2 (per 5 kg/m^2^), (I) adult height (per 5 cm), (J) ever use of oral contraceptives (yes/no), (K) current use of combined estrogen-progesterone therapy (yes/no), (L) current use of estrogen-only therapy (yes/no), (M) lifetime intake of alcohol (per 10 g/day), (N) current smoking (yes/no), (O) smoking amount (per 10 pack-years)

1. Age at menarche (per 2 years)


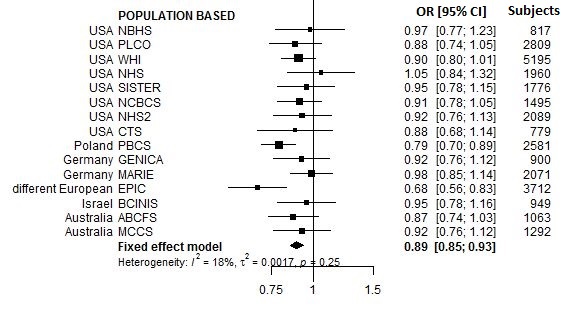


1. Parity (yes/no)


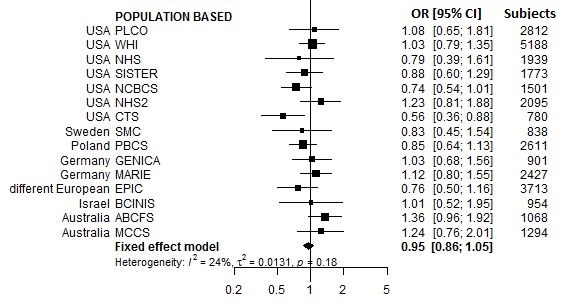


1. Number of full-term pregnancies (per pregnancy) among parous women


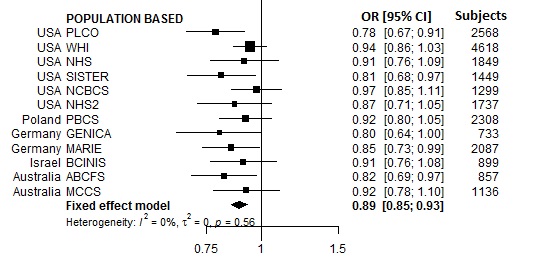


1. Ever breastfed (yes/no) among parous women


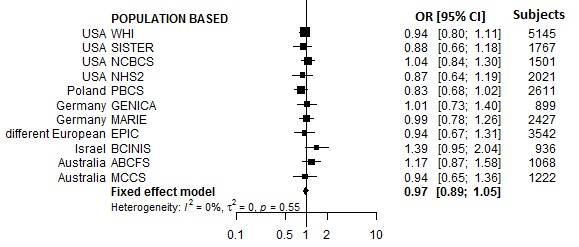


1. Duration of breastfeeding (per 12 months) among parous women


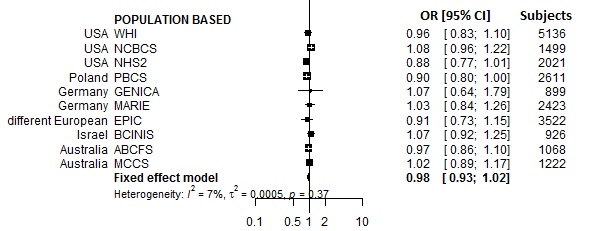


1. Age at first full-term pregnancy (per 5 years) among parous women


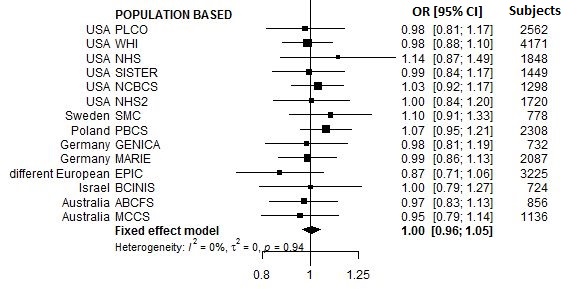


1. Adult BMI, premenopausal women (per 5 kg/m^2^)


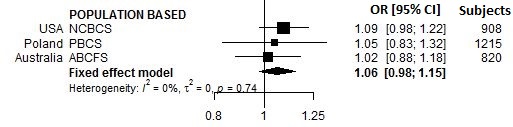


1. Adult BMI, postmenopausal women (per 5 kg/m^2^)


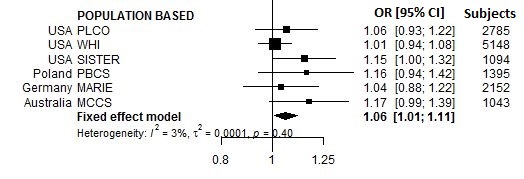


1. Adult height (per 5 cm)


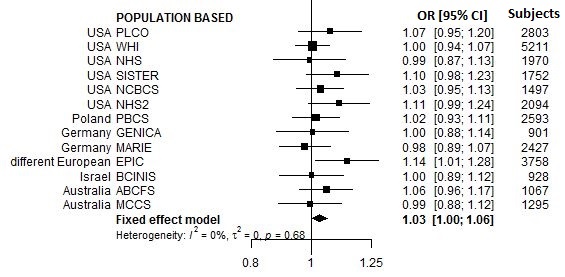


1. Ever use of oral contraceptives


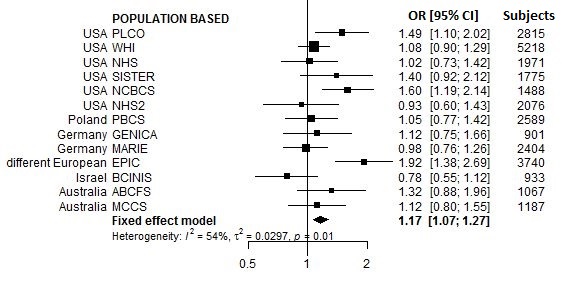


1. Current use of combined estrogen-progesterone therapy (among postmenopausal women, yes/no)


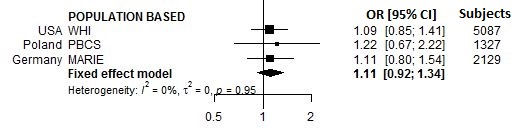


1. Current use of estrogen-only therapy (among postmenopausal women, yes/no)


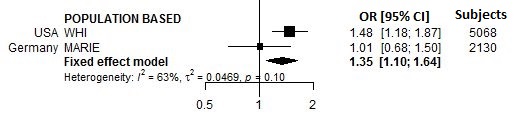


1. Lifetime intake of alcohol (per 10g/day)


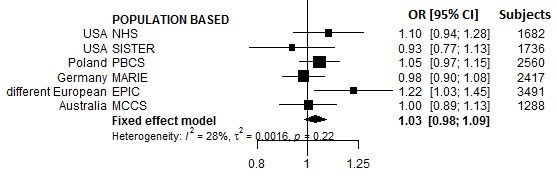


1. Current smoking (yes/no)


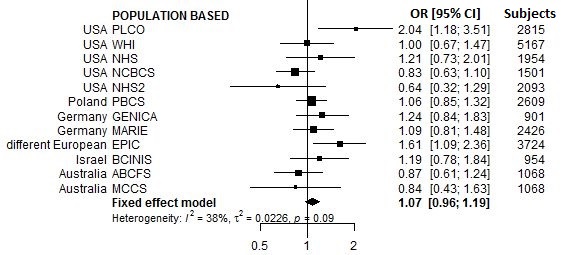


1. Smoking amount (per 10 pack-years)


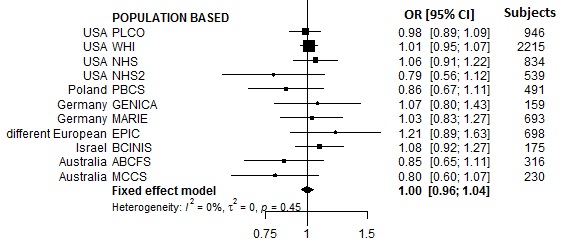

Supplement: dyz193_Supplementary_Data [file dyz193_supplementary_data.zip › dyz193-suppl_data/ije-2019-01-0030-File011.docx]
