# Supplementary material for: Assessment of interactions between 205 breast cancer susceptibility loci and 13 established risk factors in relation to breast cancer risk, in the Breast Cancer Association Consortium
Source: Int J Epidemiol. 2019 Oct 12;49(1):216–32. doi: 10.1093/ije/dyz193 (PMC7426027; doi:10.1093/ije/dyz193)
Supplement: dyz193_Supplementary_Data [file dyz193_supplementary_data.zip › dyz193-suppl_data/ije-2019-01-0030-File012.docx]

**Supplementary Figure 4.** Forest plot of meta-analyses of study-wise odds ratios and 95% confidence intervals for interactions between SNPs and environmental risk factors of breast cancer separately for OncoArray and iCOGS datasets: (A) Current use of Estrogen-Progesterone therapy × rs4442975 (Overall breast cancer risk - OncoArray dataset), (B) Current use of Estrogen-Progesterone therapy × rs4442975 (Overall breast cancer risk - iCOGS dataset), (C) Current use of Estrogen-Progesterone therapy × rs4442975 (ER-positive breast cancer risk - OncoArray dataset), (D) Current use of Estrogen-Progesterone therapy × rs4442975 (ER-positive breast cancer risk - iCOGS dataset), (E) Number of full-term pregnancies x rs6596100 (ER-negative breast cancer – OncoArray dataset), (F) Number of full-term pregnancies x rs6596100 (ER-negative breast cancer – iCOGS dataset), (G) Age at first-full-term pregnancy x rs6596100 (ER-negative breast cancer – OncoArray dataset), (H) Age at first full-term pregnancy x rs6596100 (ER-negative breast cancer – iCOGS dataset)

1. **Current use of Estrogen-Progesterone therapy × rs4442975 (Overall breast cancer risk - OncoArray dataset)**

**
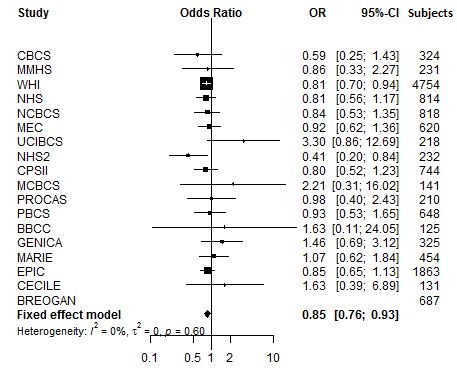
**

1. **Current use of Estrogen-Progesterone therapy × rs4442975 (Overall breast cancer risk - iCOGS dataset)**

**
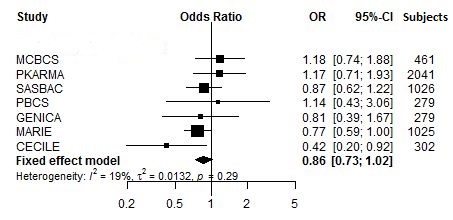
**

1. **Current use of Estrogen-Progesterone therapy × rs4442975 (ER-positive breast cancer risk - OncoArray dataset)**

**
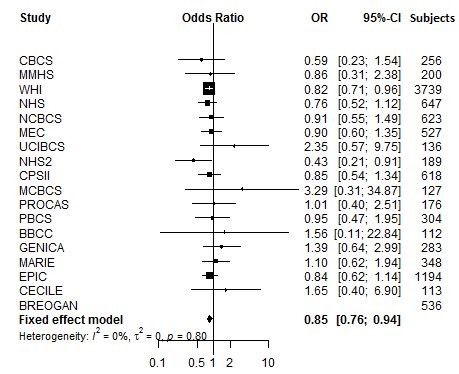
**

1. **Current use of Estrogen-Progesterone therapy × rs4442975 (ER-positive breast cancer risk - iCOGS dataset)**

**
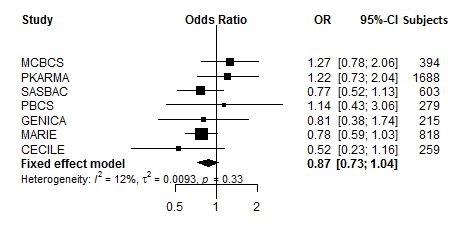
**

1. **Number of full-term pregnancies x rs6596100 (ER-negative breast cancer – OncoArray dataset)**

**
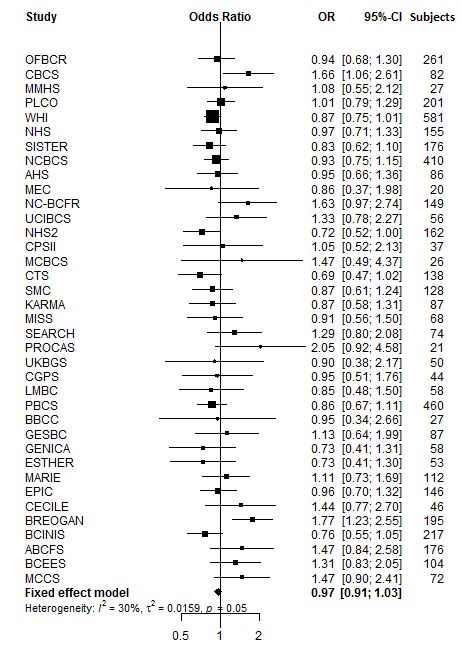
**

1. **Number of full-term pregnancies x rs6596100 (ER-negative breast cancer – iCOGS dataset)**

**
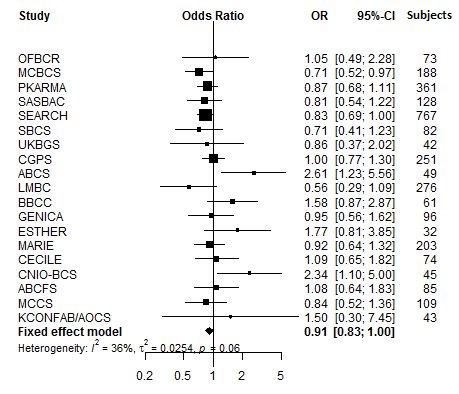
**

1. **Age at first full-term pregnancy x rs6596100 (ER-negative breast cancer – OncoArray dataset)**

**
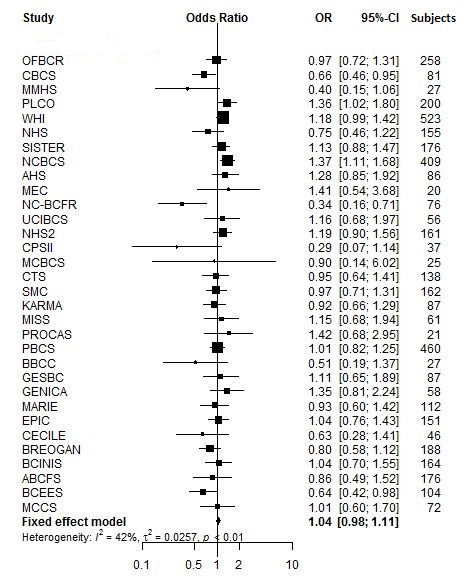
**

1. **Age at first full-term pregnancy x rs6596100 (ER-negative breast cancer – iCOGS dataset)**

**
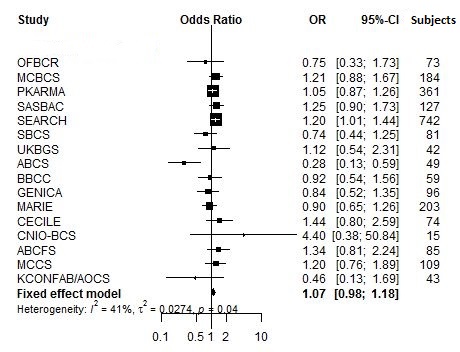
**
